# Deficits in context-dependent adaptive coding of reward in schizophrenia

**DOI:** 10.1038/npjschz.2016.20

**Published:** 2016-06-15

**Authors:** Matthias Kirschner, Oliver M Hager, Martin Bischof, Matthias N Hartmann-Riemer, Agne Kluge, Erich Seifritz, Philippe N Tobler, Stefan Kaiser

**Affiliations:** 1 Department of Psychiatry, Psychotherapy and Psychosomatics, Psychiatric Hospital, University of Zurich, Zurich, Switzerland; 2 Laboratory for Social and Neural Systems Research, Department of Economics, University of Zurich, Zurich, Switzerland; 3 Neuroscience Center Zurich, University of Zurich, Zurich, Switzerland; 4 Zurich Center for Integrative Human Physiology, University of Zurich, Zurich, Switzerland

## Abstract

Theoretical principles of information processing and empirical findings suggest that to efficiently represent all possible rewards in the natural environment, reward-sensitive neurons have to adapt their coding range dynamically to the current reward context. Adaptation ensures that the reward system is most sensitive for the most likely rewards, enabling the system to efficiently represent a potentially infinite range of reward information. A deficit in neural adaptation would prevent precise representation of rewards and could have detrimental effects for an organism’s ability to optimally engage with its environment. In schizophrenia, reward processing is known to be impaired and has been linked to different symptom dimensions. However, despite the fundamental significance of coding reward adaptively, no study has elucidated whether adaptive reward processing is impaired in schizophrenia. We therefore studied patients with schizophrenia (*n*=27) and healthy controls (*n*=25), using functional magnetic resonance imaging in combination with a variant of the monetary incentive delay task. Compared with healthy controls, patients with schizophrenia showed less efficient neural adaptation to the current reward context, which leads to imprecise neural representation of reward. Importantly, the deficit correlated with total symptom severity. Our results suggest that some of the deficits in reward processing in schizophrenia might be due to inefficient neural adaptation to the current reward context. Furthermore, because adaptive coding is a ubiquitous feature of the brain, we believe that our findings provide an avenue in defining a general impairment in neural information processing underlying this debilitating disorder.

## Introduction

Reward information is represented across several cortical and subcortical brain regions related to the dopaminergic system, such as the striatum, the orbitofrontal cortex, and the medial temporal cortices.^1–3^ Emerging evidence suggests that these representations are context specific, such that they adjust to the rewards that are available and likely in the current context.^4–7^ The dynamic adjustment in the firing of reward-sensitive dopaminergic neurons to the current context is also known as adaptive coding of reward.^8,9^ This adaptation is necessary, because the coding range of any neuron, including reward-sensitive neurons, is limited (i.e., the firing rate can increase only up to some degree and can therefore resolve small input differences only with limited precision), whereas the diversity and range of potential inputs, including rewards, in our daily life is theoretically unlimited. Hence, if the full neuronal coding range would always be devoted to represent all possible inputs, e.g., reward amounts, only relatively large differences in these inputs could be represented and discrimination between different levels would be imprecise. Although in principle the issue could be mitigated somewhat by increasing the number of recruited neurons with increasing reward amounts, the problem remains that for each neuron small amount differences can be resolved only with limited precision.

**Figure 1:**
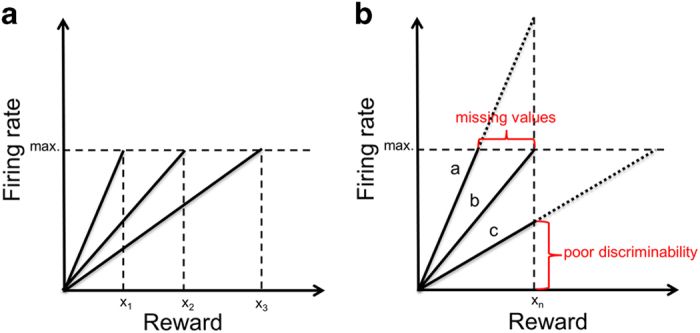
A simple model of efficient and inefficient adaptive coding. (**a**) A simple model of adaptive coding of reward. To efficiently encode all possible rewards with a limited coding range, the brain is dynamically adjusting the response sensitivity to the currently available rewards. This mechanism allows an optimal discrimination between different amounts of reward in any given context, enabling efficient processing of reward information. (b) Contrast of optimal and disturbed adaptive coding. This plot illustrates two potential consequences of inefficient adaptation of the coding range. Response function (a) is too steep, leading to a miscoding/incomplete representation of reward information. Response function (c) is too shallow, which leads to poor discriminability of reward due to a restricted coding range. Response function (b) shows optimal adaptive reward coding, where the slope of the response function adapts so as to represent the full range of reward.

An efficient solution to this problem has been characterized both for sensory and reward systems. It consists of adapting the sensitivity of neurons to the range of sensory or reward inputs that are most likely in the current context.^9,10^ As a result, in case of reward-coding dopamine neurons, the slope of the neuronal response function is steeper in contexts with smaller reward ranges and shallower in contexts with larger-reward ranges (Figure 1a).^11^ Thus, adapting slopes ensure optimal sensitivity to currently available and likely rewards. This mechanism allows the organism to discriminate between different reward amounts as well as possible, and thereby enables optimally informed behavior. On the other hand, if there is no adaptation or if the adaptation is inefficient, reward information cannot be fully encoded, resulting in a loss of information and therefore in uncertainty about the precise reward amount of a stimulus or an action.^12^ This uncertainty can be caused by two different mechanisms: in case the response function is too narrow, reward amounts at the extreme end of the distribution would be misrepresented. In case the response function is too wide, it would result in unnecessarily flat slopes with poor discrimination between different reward amounts (Figure 1b). In summary, a lack of dynamic neural adaptation leads to uncertainty in the representation of reward, which might result in detrimental decision-making and a failure to efficiently engage with the environment.

Dysfunction in dopamine transmission is a central feature of schizophrenia (SZ) and has been linked to various deficits in reward processing, such as impairments in reinforcement learning,^13,14^ reward anticipation,^15–17^ cost–benefit computation,^18,19^ and aberrant salience coding.^20–22^ However, despite the significance of adaptive coding as a fundamental solution for the problem of sensitivity in representing reward information, to our knowledge no study has investigated whether dynamic neural adaptation to available rewards is deficient in SZ. We therefore constructed a modified version of the monetary incentive delay task that enabled us to investigate adaptive coding of reward during the outcome phase. We hypothesized that patients with SZ show deficits in neural adaptation in reward-coding regions, such as the striatum, in comparison with healthy controls (HCs). Furthermore, we explored whether the degree in adaptive reward coding is associated with symptom severity.

## Results

### Reward increases response time across groups

Participant demographics, clinical data, and group comparisons are summarized in Table 1. Regarding response time, the repeated measures analysis of variance revealed no significant main effect of group (F(1,50)=2.91, *P*=0.09), but a significant main effect of reward context (F(1,50)=36.2, *P*<0.0001). Across all participants, response times were faster in the high-reward context, indicating that participants adapted their behavior to the different reward context (low versus high reward). There was no significant group by reward interaction effect (F(1,50)=0.65, *P*=0.47). Due to low error rates, we used a total error score for group comparison and did not find any group differences (HC=3.3 (2.5); SZ=3.8 (2.8.); *U*=315, *P*=0.68). Finally, both groups differed significantly in total win (HC=38.9 (5.2); SZ=36 (4.6); *t*=2.1, *P*=0.04), although the differences were small.

### Significant group differences in adaptive coding of reward

To assess reward adaptation at the neural level, we first identified brain regions coding reward amount. Voxel-wise whole-brain analysis of parametrically increasing responses across all subjects during reward outcome ((parametrically modulated (pmod) low reward)+(pmod high reward)) revealed several brain regions sensitive for reward amount (cluster-level family-wise error (FWE) corrected, *P*⩽0.05), i.e., caudate, putamen, medial orbitofrontal cortex, and anterior insula/inferior frontal gyrus (Supplementary Table S1). Thus, activation in these regions increases with reward amount at the time of outcome.

In our paradigm, adaptive coding corresponds to a steeper response slope in the low-reward condition than in the high-reward condition (Figure 1a; Experimental design and task). We therefore investigated adaptive coding with the contrast ((pmod low reward)−(pmod high reward)) within the reward-sensitive regions identified in the previous analysis (one single ROI including all significant clusters described in Supplementary Table S1). In healthy subjects, this contrast revealed several significant clusters, including caudate, cingulate cortex, insula, superior frontal gyrus, and precuneus (FWE corrected, *P*<0.05), indicating wide-spread implementation of adaptive coding principles in the reward system (Supplementary Table S2). By contrast, no significant effect for the adaptive coding contrast was found in patients with SZ.

**Figure 2:**
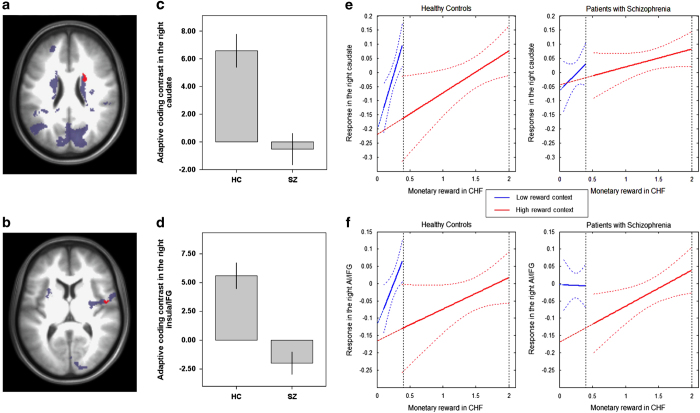
Reward-sensitive regions showing group differences in adaptive coding. Brain regions responding to reward amount and showing differential adaptive coding. Reward responses ((pmod low reward)+(pmod high reward)) are displayed in blue. In red are clusters, where healthy controls showed significantly stronger activation increases in the adaptive coding contrast ((pmod low reward)−(pmod high reward)). Brain images thresholded at *P*<0.05 (FWE). (**a**) Axial image of the right caudate. (**b**) Axial image of the right insula/IFG. Columns in bar graphs illustrate red clusters and reflect adaptive coding contrast estimates ((pmod low reward)−(pmod high reward)) for each group separately (**c**,**d**). Response functions of the neural adaptation in the right caudate (**e**) and the right anterior insula/inferior frontal gyrus (**f**) plotted separately for the low-reward (blue) and the high-reward (red) context. For visualization purposes, each reward context was divided in two mean reward levels (low reward=CHF 0–0.2, CHF 0.2–0.4; high reward=CHF 0–1, CHF 1–2), which is represented by the *x* axis. The *y* axis represents the adaptive coding contrast estimates ((pmod low reward)−(pmod high reward)). Healthy controls optimally adapt the coding range to the current range of rewards in both regions, resulting in a steeper slope of neural responses in the low-reward context than in the high-reward context (**e**,**f**; left). In contrast, patients with schizophrenia show significant deficits in adaptive coding, with insufficient slope increase (caudate; **e**, right) or even shallower slope (insula; **f**, right) for the low-reward context compared with the high-reward context. The diminished steepness of slopes translates to reduced discriminability in both reward contexts for the right caudate of patients. By contrast, in the right AI/IFG, discriminability was reduced primarily in the low-reward context, whereas it was comparable to the discriminability of healthy controls in the high-reward context. AI, anterior insula; FWE, family-wise error; IFG, inferior frontal gyrus; pmod, parametrically modulated.

Importantly, we tested for group differences in adaptive coding between HCs and patients with SZ in the same single ROI consisting of all reward-sensitive regions (all significant clusters described in Supplementary Table S1). Specifically, we compared the slope differences in the reward response functions for low and high reward ((HC (pmod low reward)−(pmod high reward))−(SZ (pmod low reward)—(pmod high reward))). In the caudate (*x*=21, *y*=3, *z*=22; cluster size=19, *t*=4.7, FWE corrected *P*⩽0.05; Figure 2a,c) and the anterior insula/inferior frontal gyrus (*x*=50, *y*=−4, *z*=6; cluster size=24, *t*=5.43, FWE corrected *P*⩽0.05; Figure 2b,d), HCs showed significantly stronger adaptive coding ((pmod low reward)−(pmod high reward)) relative to patients with SZ. The results were similar when we used an anatomically defined ROI of the striatum defined with the Wake Forest University Pickatlas Toolbox in SPM8^23^ (right: *x*=23, *y*=2, *z*=21, cluster size=39; left: *x*=−6, *y*=14, *z*=12, cluster size=8, both FWE corrected *P*⩽0.05). To visualize these differences in the adaptive coding of reward, we plotted the response functions of the neural activity in the low- and high-reward context separately for the two groups (Figure 2e,f). These findings imply significant differences between the slope of the response function in the low- and high-reward context in HCs, but not in patients with SZ. In other words, these significant group differences provide strong evidence for efficient neural adaptation of reward coding in the caudate and anterior insula/inferior frontal gyrus in HCs, but not in the patient group.

Next, we tested whether there were any clusters in the reward-sensitive regions. where patients with SZ showed more efficient adaptive coding of reward than HCs ((SZ (pmod low reward)−pmod high reward))−(HC (pmod low reward)−(pmod high reward))). In this analysis, we found no significant activation at the previously used more stringent threshold (FWE corrected, *P*⩽0.05) and even at a more lenient threshold (*P*<0.001, uncorrected, extent threshold >10 voxels).

### Deficits of adaptive coding correlate with general symptomatology

**Figure 3:**
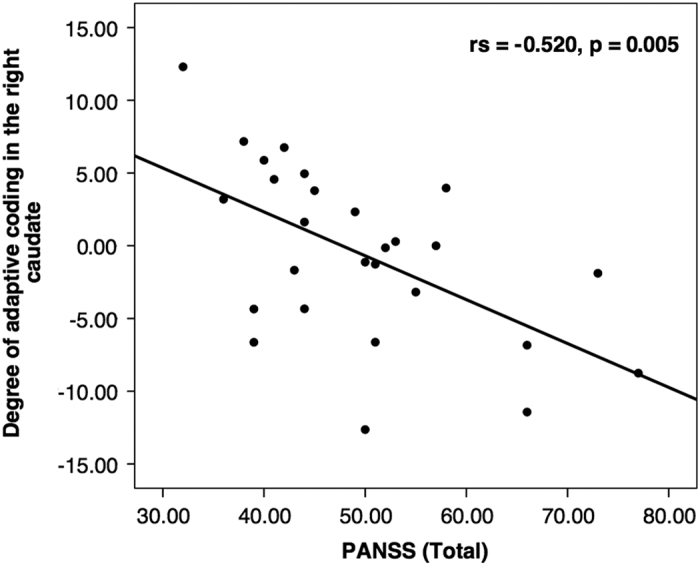
Correlation plot of adaptive coding with symptom severity. Correlation plot of the adaptive coding contrast estimates ((pmod low reward)−(pmod high reward)) with the PANSS total score in patients with schizophrenia. Contrast estimates were extracted from the caudate region showing significant group differences (red cluster in Figure 2a). In the right caudate, we found a significant negative correlation of the degree of adaptive coding with the PANSS total score. PANSS, Positive and Negative Syndrome Scale; pmod, parametrically modulated.

To test whether individual differences in the degree of adaptive coding relate to symptom severity in patients with SZ, we used the individual contrast estimates ((pmod low reward)−(pmod high reward)) extracted from the regions showing significant group differences in adaptive coding. Correlation analysis showed that reduced adaptive coding in the right caudate correlated with symptom severity as determined with the Positive and Negative Syndrome Scale (PANSS) total score (*r*_s_=−0.520, *P*=0.005, Bonferroni adjusted *P*=0.020), reflected in a highly significant negative correlation in the patient group (Figure 3). Importantly, this effect was also present for positive symptoms (PANSS-positive symptom score; *r*_s_=−0.466, *P*=0.014), negative symptoms (PANSS-negative symptom score; *r*_s_=−0.529, *P*=0.005) and general symptoms (PANSS general symptom score; *r*_s_=−0.428, *P*=0.026). Furthermore, we found a trend effect of a negative correlation between reduced adaptive coding in the caudate and the Brief Negative Symptom Scale (BNSS) total score (*r*_s_=−0.350, *P*=0.073, Bonferroni adjusted *P*=0.292)). However, there were no significant correlations with the two negative symptom factors apathy (*r*_s_=−0.288, *P*=0.145) and diminished expression (*r*_s_=−0.292, *P*=0.139). In addition, we computed a partial correlation to account for medication effects (chlorpromazine equivalents) and cognition (composite cognition score), which remained highly significant for the PANSS total score (*r*_s_=−0.539, *P*=0.005) and yielded a significant correlation with BNSS total score (*r*_s_=−0.401, *P*=0.047). Thus, the observed correlations were not decreased when controlling for medication and cognition.

The correlation analysis between adaptive coding contrast estimates ((pmod low reward)−(pmod high reward)) in the anterior insula/inferior gyrus and symptom severity yielded no significant correlation with the PANSS total score (*r*_s_=−0.223, *P*=0.263, Bonferroni adjusted *P*=1.052) and BNSS total score (*r*_s_=−0.017, *P*=0.935, Bonferroni adjusted *P*=3.812).

Finally, we did not find any significant association of the adaptive coding contrast ((pmod low reward)−(pmod high reward)) with potential confounding variables such as chlorpromazine equivalents (right caudate: *r*_s_=−0.076, *P*=0.708; insula/inferior frontal gyrus: *r*_s_=−0.142, *P*=0.481) or cognition (right caudate: *r*_s_=−0.121, *P*=0.546; insula: *r*_s_=−0.016, *P*=0.935). Concerning possible associations with behavior, total win was not associated with reduced adaptive coding (SZ, right caudate: *r*_s_=−0.213, *P*=0.286; insula/inferior frontal gyrus: *r*_s_=0.054, *P*=0.788; HC, right caudate: *r*_s_=−0.076, *P*=0.718; insula/inferior frontal gyrus: *r*_s_=−0.232, *P*=0.265). In sum, higher symptom severity was associated with a greater deficit in adaptive coding in the caudate. Furthermore, this deficit in adaptive coding was not related to medication dose, cognition, or differences in task performance.

## Discussion

In the current study, we used a modified version of the monetary incentive delay paradigm to investigate adaptive coding of reward for the first time in patients with SZ. Patients with SZ show inefficient adaptive coding in two reward-sensitive regions, namely, in the right caudate and the right anterior insula/inferior frontal gyrus. These findings imply that in patients with SZ, these two regions fail to take advantage of contextual information to adjust their sensitivity to more likely amounts, which would normally allow for a precise and efficient representation of reward information. Furthermore, the deficit in adaptive coding is related to the total symptom severity of patients with SZ. Particularly in the right caudate, the impairment in adaptive coding correlated with psychopathological measures (PANSS total score, PANSS subscales, and BNSS total score). At the behavioral level, we observed group differences in total amount of money won. However, these behavioral differences were not correlated with deficits in adaptive coding. When interpreting this finding, several points need to be kept in mind. (1) The observed differences in total win between HC and SZ were small. (2) The differences in total win may be related not only to adaptive coding deficits. (3) The degree of adaptive coding deficits may be a more important factor in different behavioral situations, e.g., when small reward amount differences need to be discriminated in amount-based choice. (4) Compensating mechanisms may help to reduce the impact of adaptive coding deficits on the behavioral outcome (note that also non-adaptive reward amount coding maintains basic sensitivity to reward amount). In agreement with these points, one recent functional magnetic resonance imaging study showed neural adaptation without behavioral effects in healthy subjects.^5^

Overall, the present findings suggest that patients with SZ have deficits in adaptive coding of rewards due to a diminished discriminability of different reward amounts, which leads to an imprecise representation of reward information. In addition, our results imply that this deficit is related to the overall symptom severity.

These findings substantially expand the current understanding of reward-processing deficits in SZ by introducing a new concept—the contextual adaptation of neural sensitivity to reward. We provide evidence that patients with SZ show significant deficits in adaptive coding of reward compared with HCs in two reward-sensitive regions, the right caudate and the right anterior insula/inferior frontal gyrus. In the right caudate, patients only exploit a fraction of the response range to represent reward information compared with HCs, which severely impairs their ability to discriminate between different reward amounts. The caudate, together with the putamen, forms the dorsal part of the striatum, which is involved in reward-guided action selection and in learning about actions and their reward consequences.^24–29^ Our findings of an adaptive coding deficit is in line with work by Morris *et al.*^30^ who recently described an association of caudate dysfunction with an impairment in integrating changes in experienced reward values (devaluation of food rewards) to guide choice behavior in patients with SZ.

In the anterior insula/inferior frontal gyrus, adaptation in the low-reward context was so disrupted in patients that reward amount was no longer encoded with a positive slope. However, reward amount in the high-reward context was encoded similarly to HCs, suggesting partly preserved sensitivity to reward. The observed deficits in adaptive coding in the anterior insula and the related structure of the inferior frontal gyrus are in line with aberrant salience processing observed in SZ.^31–35^ Specifically, the imprecise neural representation of reward information could lead to increased uncertainty about external stimuli or internal values, which in turn may alter the processing of what is important and subsequently lead to the attribution of aberrant salience. In support of this notion, recent findings suggest that reward uncertainty enhances salience attribution.^36,37^

Furthermore, we show that the adaptive coding deficit is related to total symptom severity, suggesting that this deficit could reflect a more basic dysfunction instead of a specific neural correlate of positive, negative, or depressive symptoms.^38–41^ The idea of a general dysfunction is in line with the ubiquity of a context-dependent adaptation of dopamine neurons in the human brain.^9,11^ An imprecise encoding of reward information could potentially have far-reaching consequences, because precise reward information can be considered a prerequisite for all further reward-related processes. With regard to the idea of a general dysfunction, it is also important to bear in mind that a context-dependent adaptation of the neural activity does not solely apply to the encoding of reward information, but also to sensory information processing, i.e., the processing of auditory and visual information.^42–44^ Furthermore, earlier studies have proposed that the ‘core’ cognitive deficit in SZ is a disturbance of context information processing.^45–47^ However, to support the hypothesis of a general deficit in a context-dependent neural adaptation, this principle would have to be studied in the sensory and cognitive domains in addition to the reward domain.

### Limitations and future directions

As all patients were treated with second-generation antipsychotics, we cannot exclude an impact of treatment on the group comparison. However, within the patient group, antipsychotic dose was not related to adaptive coding and the association of adaptive coding with symptoms remained significant when controlling for antipsychotic dose. This suggests a limited impact of antipsychotic treatment and D2 blockade on adaptive coding, but studies with unmedicated patients would be very valuable. In addition, other neurotransmitters such as glutamate may have a role in adaptive coding of reward. Indeed, White *et al.*^48^ showed an association between glutamate in the substantia nigra and reward processing in HC. Importantly, they suggested that elevated glutamate levels in SZ are linked to aberrant prediction error signals.^48^ It is tempting to speculate that recently described glutamatergic abnormalities in the striatum may contribute to adaptive coding deficits.^49^ Therefore, future multimodal imaging studies are needed to elucidate the role of glutamatergic abnormalities and reward-processing deficits in SZ.

Another limitation of the study is related to the fact that our sample showed relatively low levels of positive and depressive symptoms, which limits the possibility to differentiate specific effects of these domains. Although current substance use disorders and current illegal drug use were exclusion criteria, we did not apply objective drug tests (e.g., urine or hair samples). Future studies should investigate the impact of drug use on adaptive coding in HCs and patients. Last, it has to be mentioned that due to the task design, it is difficult to precisely disentangle reward anticipation and reward outcomes. In the present study, this limitation holds for the mean outcome regressors, which we did not analyze. However, thanks to the parametric variation of the task design, the parametric outcome regressors of interest were not correlated with the anticipation regressor.

### Conclusion

In summary, the present findings provide the first evidence that patients with SZ show deficits in adaptive coding of reward. Insufficient adaptation causes imprecise representation of reward information due to diminished discriminability of different reward amounts. This has broad potential impact on reward-related processes, which is in line with our observation that deficient adaptive coding is related to total symptom severity. Finally, we believe that our findings contribute to a better understanding of the reward-processing deficits in SZ and suggest a new approach to identify a general impairment in neural mechanisms underlying this debilitating disorder.

## Materials and methods

The presented analysis for adaptive coding during reward outcomes rely on the functional magnetic resonance imaging data set previously used in the study by Kirschner *et al.*^16^ The previous analysis focused only on the classical binary contrast between reward and no-reward anticipation at the stimulus presentation phase. Here we take advantage of our advanced task design and present an entirely different and novel analytical approach specific to adaptive coding of reward amounts at the outcome phase using parametric contrasts. Although this approach is orthogonal to the previous approach, we fully control for stimulus presentation effects.

### Participants

We acquired the data from 29 patients with SZ and 28 HC participants. Participants with SZ were recruited from outpatient (*n*=12) and inpatient (*n*=17) units of the Psychiatric Hospital of the University of Zurich or from affiliated institutions. All patients with SZ were clinically stable and received a stable dose of medication (no change at least for 7 days before testing). Definition of clinical stability in inpatients included that they were at the end of their hospitalization, participated in a multimodal treatment program, and engaged in activities outside the hospital. Please note that in Switzerland, the average duration of inpatient treatment of patients with SZ is ~40 days,^50^ which means that nearly all of our inpatients would have been treated as outpatients in other health-care systems.

The project was approved by the local ethics committee. All participants gave written informed consent to participate in the study according to the Declaration of Helsinki. Diagnosis of SZ was confirmed in a structured Mini-International Neuropsychiatric Interview for the 4th edition of the Diagnostic and Statistical Manual of Mental Disorders (DSM-IV).^51^ We excluded participants with any other DSM-IV axis I disorder (in particular current substance use disorders), medication with lorazepam >1 mg, acute psychotic symptoms, i.e., any positive subscale item score higher than four as measured with the PANSS,^52^ and extrapyramidal side effects, i.e., a total score higher than two on the Modified Simpson-Angus Scale.^53^

All study participants underwent an extensive psychopathological assessment. Severity of positive and negative symptoms was assessed with the PANSS. Negative symptoms were specifically assessed with the BNSS.^54^ Details on further psychopathological and neuropsychological assessment can be found in the Supplementary Methods.

### Experimental design and task

**Figure 4:**
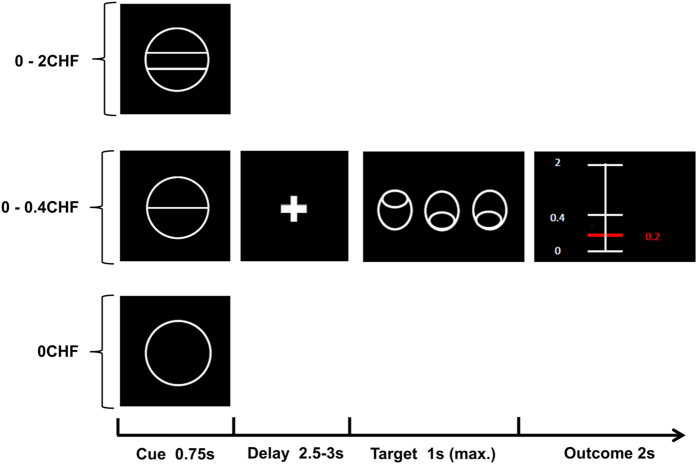
Task design of the adapted monetary incentive delay task. Adapted monetary incentive delay task: at the beginning of each trial, one of three different cues was presented for 0.75 s. The cue indicated the reward context, specifically the range of possible amounts participants could gain in that trial, i.e., 0–2 Swiss Francs (CHF; circle with two lines), CHF 0–0.40 (circle with one line), or CHF 0 (circle only; at time of testing CHF 1=USD 1.08). After a delay, varying from 2.5 to 3 s, the participants had to identify an outlier from three presented circles and press a button (either left or right) as fast as possible (varying from 0.32 to 1 s). In case of a correct answer, participants were immediately notified of the amount of money they had won, which directly depended on their individual task performance (duration of feedback 2 s). The gain of each trial was calculated based on the response times of the previous 15 individual trials. Error trials were defined as trials with a wrong response or late response (>1 s) and participants did not receive any monetary reward.

We used a variant of the monetary incentive delay task^55^ with stimuli based on the cued-reinforcement reaction time task used by Cools *et al*.^56^ (Figure 4). This variant enabled us to investigate reward anticipation and reward outcome separately. In each correct trial, participants received a reward, which was determined directly by the individual response time (for further details, see Supplementary Methods and Supplementary Figure S1). Thus, in contrast to most versions of the monetary incentive delay task, there was no dichotomy reward/no-reward in the outcome phase, but a continuous distribution of rewards, which allowed us to study reward amount processing separately from reward anticipation. Importantly, our task included two different reward contexts, a low-reward context, ranging from Swiss franc (CHF) 0 to 0.4, and a high-reward context, ranging from CHF 0 to 2.0 (in addition to a neutral control condition without reward). The differential reward range of the low- and high-reward context allowed us to investigate the dynamic adaptation of reward activation in the current reward context. In particular, adaptation would correspond to a steeper slope of the mapping between output (response strength) and input (reward amount) for the low-reward context compared with the high-reward context (Figure 1a and below).

Before starting the experiment, we informed all participants that they would receive the accumulated amount of money they won during the two experimental sessions. The maximum amount of money to be won was CHF 50. Every participant performed two training runs, one outside and one inside the scanner. Excluding the training sessions, the experiment contained two runs with 36 trials each, resulting in 24 trials per reward condition, with every trial lasting ~10 s. The intertrial interval was jittered from 1 to 9 s with a mean of 3.5 s. In total, one run lasted ~6 min. The task was implemented using the MATLAB toolboxes Cogent 2000 (http://www.vislab.ucl.ac.uk/cogent.php, developed at the FIL and the ICN, UCL, London, UK) and Cogent Graphics (http://www.vislab.ucl.ac.uk/cogent.php, developed at the LON, UCL, London, UK). For acquisition parameters, see Supplementary Methods.

### Data analysis

All demographic, clinical, neuropsychological, and behavioral data, as well as the correlations were analyzed using IBM SPSS Statistics Version 22 (SPSS Inc., Chicago, IL, USA). We analyzed the functional magnetic resonance imaging data using SPM8 (Statistical Parametric Mapping, Wellcome Department of Cognitive Neurology, London, UK).

### Behavioral data analysis

The main behavioral outcome measure was response time, defined as time between target presentation and pressing the correct answer button. We performed a two-way repeated measures analysis of variance with the group as between-subject factor and reward context (low and high) as within-subject factor. Potential group differences in all other behavioral data were investigated using two-tailed two-sample *t*-tests. For non-normally distributed data (as assessed by the Kolmogorov–Smirnov test), Mann–Whitney *U*-tests were applied.

### Image preprocessing

Functional images were corrected for differences in the time of slice acquisition. The Realign and Unwarp functions of SPM8 were used to correct our data for head motion. A voxel displacement map, calculated from double phase and magnitude field map data, was used to correct for combined static and dynamic distortions. We performed segmentation, bias correction, and spatial normalization. Finally, images were smoothed using a Gaussian kernel of 6-mm width at half maximum. We evaluated the quality of functional magnetic resonance imaging data by manual inspection and excluded data with poor quality due to significant signal dropout in echo planar image (EPI) sequences (one patient with SZ). Furthermore, participants with translational head movement >3 mm (1-voxel size) were excluded (five participants: 3 HC and 2 SZ), leaving a total sample of 27 patients with SZ and 25 HCs. For group comparison of head movement, we calculated the mean relative displacement (MRD) according to the approach of van Dijk *et al.*^57^ and Satterthwaite *et al*.^58^ Mean MRD did not differ between HCs and patients with SZ (HC: MRD=0.11, s.d.=0.03; SZ: MRD=0.11, s.d.=0.04, *P*=0.43).

### First-level image analyses

We computed a general linear model with a parametric design to identify brain regions that encode reward amount in an adaptive manner at the outcome phase. In particular, we modeled each reward condition separately (no-/low-/high-reward outcome). Please note that these regressors accounted for potential differences in mean activation between the low- (CHF 0–0.4) and the high-reward context (CHF 0–2.0). Importantly, the low- and high-reward outcome regressors were pmod by the actual outcome won in each trial (pmod low reward, pmod high reward). These modulators capture reward amount and are orthogonal to the mean regressors; pmod low ranged from CHF 0 to 0.4, whereas pmod high ranged from CHF 0 to 2.0, resulting in a smaller range for the low-reward outcomes. One regressor for the anticipation phase, one regressor for target presentation, and one regressor for error trials were modeled as regressors of no interest. In total, the first-level model included eight regressors. The canonical hemodynamic response function was used for convolving all explanatory variables. Please note that by design in this model the two parametrically modulated reward regressors of interest are not correlated with the anticipation regressor, which serves to account for visual activations due to stimulus presentation.

### Second-level image analyses: identification of reward-sensitive regions

At the second (i.e., group) level of analysis, we included the individual contrast images of all participants in a random-effects model. We assessed within-group activation using one sample *t*-tests and between-group activation using two-sample *t*-tests. To identify brain regions coding reward amount, we used a contrast including both parametric modulators ((pmod low reward)+(pmod high reward)), which we applied in a voxel-wise whole-brain analysis across all participants. The statistical threshold was set to *P*⩽0.05, whole-brain cluster-level FWE rate corrected for multiple comparisons, with a cluster-inducing voxel threshold of *P*<0.001.

### Second-level image analyses: adaptive coding of reward

In a second step, we tested adaptive coding of reward within the previously identified reward-sensitive brain regions, using one single ROI including all significant clusters. Efficient neural adaptation of reward implies that the responses dynamically adjust to the range of possible rewards. Therefore, the slope of the response function should be steeper with a smaller reward range compared with a larger-reward range (Figure 1a). Consequently, in case of adaptive coding in our task, the slope of the response function in the low-reward context should be steeper than the slope in the high-reward context. To test for a significant difference, we therefore subtracted the beta estimates of the high-reward parametric regressor from those of the low-reward parametric regressor ((pmod low reward)−(pmod high reward)). A significant result within the identified reward regions provides strong evidence for adaptive reward coding, because it reflects a significant difference in the slope of the reward response function.

First, we investigated adaptive coding of reward in HCs and patients with SZ separately. Second, based on our main hypothesis that patients show less adaptive coding than HCs, we computed group differences between HCs and patients with SZ in the adaptive coding contrast ((HC (pmod low reward)−(pmod high reward))−(SZ (pmod low reward)−(pmod high reward))). Please note that this contrast is independent of the one used to identify the reward-related ROIs. Finally, we evaluated if efficient adaptation of reward is correlated with symptom severity in patients with SZ. For this purpose, we extracted the adaptive coding contrast estimate ((pmod low reward)−(pmod high reward)) for the regions showing significant group differences in adaptive coding. We then performed Spearman’s rank correlation analyses between the adaptive coding contrast estimate and symptom severity ratings. These main analyses with both total symptom scores (PANSS total score and BNSS total score) were corrected for multiple comparisons using Bonferroni correction.

## Figures and Tables

**Table 1:** Demographic, psychopathological, and clinical data

	*Patient group (*n*=27)*	*Healthy controls (*n*=25)*	*Test statistic (*t*/*Χ^*2*^*/*U*)*	P* value*
Age	31.9 (7.1)	33.0 (9.7)	*U*=322.00	0.78
Gender (female/male)	9/18	9/16	*Χ*^*2*^=0.04	0.81
Education in years	12.2 (3.0)	12.4 (3.6)	*U*=334.00	0.95
Duration of illness in years	9.2 (6.6)	—	—	—
Age of onset in years	22.7 (6)	—	—	—
Chlorpromazine equivalents (mg/d)	491.3 (349.5)	—	—	—
				
*Psychopathology*				
PANSS positive	11.2 (2.9)	—	—	—
PANSS negative	14.7 (5.8)	—	—	—
PANSS general psychopathology	23.5 (4.8)	—	—	—
PANSS total	49.4 (11.2)	—	—	—
BNSS apathy^a^	14.8 (6.9)	—	—	—
BNSS diminished expression^b^	8.5 (7.2)	—	—	—
BNSS total	24.6 (12.4)	—	—	—
CDSS Total	1.6 (2.2)	—	—	—
GAF	56.9 (9.6)	—	—	—
				
*Cognition*^c^				
Composite cognition score	−0.62 (0.89)	0 (0.53)	*t*=3.0	0.01*
MWT IQ	25.9 (5.8)	27.6 (4.0)	*t*=1.2	0.23

Abbreviations: BNSS, Brief Negative Symptom Scale; CDSS, Calgary Depression Scale for Schizophrenia; GAF, Global Assessment of Functioning; MWT IQ, Multiple Word Test Intelligence Quotient; PANSS, Positive and Negative Syndrome Scale.

Note: Data are presented as means and s.d.’s. Potential group differences were investigated using two-sample *t*-tests for continuous and *χ*^2^-tests for categorical data. For non-normally distributed data Mann–Whitney *U*-tests were applied. All patients were receiving atypical antipsychotics at the time of testing.

a Apathy=avolition, anhedonia, asociality.

b Diminished expression=affective flattening or blunting, alogia.

c Cognition data were z-transformed based on the data of the HC group for each test separately. The Composite cognition score was computed as the mean of the z-transformed test scores on subject level.

*P<0.05.
