# Supplementary material for: Deficits in context-dependent adaptive coding of reward in schizophrenia
Source: NPJ Schizophr. 2016 Jun 15;2:16020–. doi: 10.1038/npjschz.2016.20 (PMC4945098; doi:10.1038/npjschz.2016.20)
Supplement: Supplementary Information [file npjschz201620-s1.doc]

**Deficits in context-dependent adaptive coding of reward in schizophrenia**

*Supplemental Information*

1. Supplementary Methods
   1. Psychopathological and neuropsychological assessment
   2. Functional imaging data acquisition
   3. Figure S1. Graphical illustration of pay-out structure
2. Supplementary Results
   1. Table S1. Whole brain analyses of reward coding regions across all subjects
   2. Table S2. Within-group analysis of adaptive coding in healthy controls
3. References
4. Supplementary Methods
   1. Psychopathological and neuropsychological assessment

All study participants underwent an extensive psychopathological assessment. Severity of positive and negative symptoms was assessed with the PANSS. Negative symptoms were specifically assessed with the brief negative symptom scale (BNSS).^1^ Further psychopathological assessment included the Calgary Depression Scale for Schizophrenia (CDSS), the Global Assessment of Functioning scale (GAF), and the Personal and Social Performance scale (PSP).^2–4^ Moreover, both groups completed a neuropsychological test battery assessing verbal learning (Auditory Verbal Learning Memory Test; ^5^, verbal and visual short-term working memory (digit span; Corsi block-tapping test)^6,7^, processing speed (Digit-Symbol Coding)^8^, planning (Tower of London)^9^, and semantic and phonetic fluency (animal naming, s-words).^10^ Results of all cognitive tests were summarized in a composite cognition score computed with the mean of z-transformed scores (based on HC group data). Additionally, we used the Multiple Word Test^11^ to control for premorbid verbal intelligence.

- 1. Functional imaging data acquisition

Imaging data was collected with a Philips Achieva 3.0T magnetic resonance (MR) scanner using a 32 channel SENSE head coil (Philips, Best, The Netherlands) at the MR center at the Psychiatric Hospital, University of Zurich. Functional MRI (fMRI) was acquired in two runs with 195 ascending transverse plane images using a gradient-echo T2*-weighted echo-planar image (EPI) sequence over the whole brain. Acquired in-plane resolution was 3×3mm2, 3mm slice thickness and 0.5mm gap width over a field of view of 240×240mm2, a repetition/echo time (TR/TE) of 2000/25ms and a flip angle of 82°. The first five scans were discarded to eliminate the influence of T1 saturation effects. Slices were aligned with the anterior–posterior commissure. Anatomical data was acquired with an ultrafast gradient echo T1-weighted sequence in 160 sagittal plane slices of 240×240mm2 resulting in 1x1x1mm3 voxels.

- 1. Figure S1. Graphical illustration of pay-out structure. Adapted from Kirschner et al. ^12^


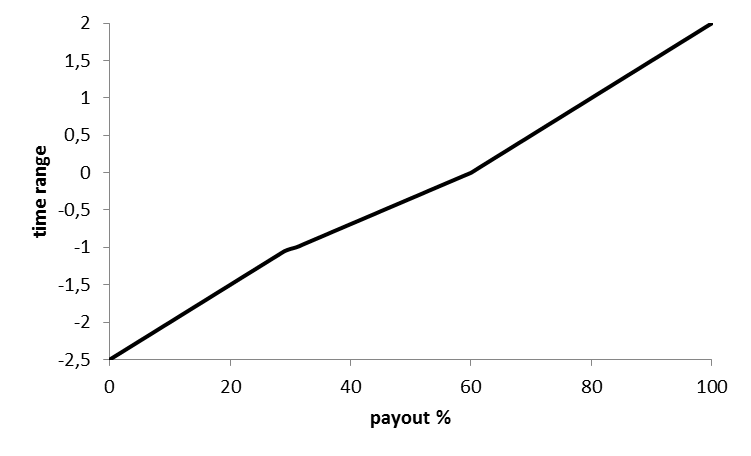


Pay-out structure of the Monetary Incentive Delay task variant. For every individual, we calculated the 15 previous response times and sorted them from fast to slow using a simple bubble sorting procedure. We then selected the response times corresponding to the 60^th^ percentile and 80^th^ percentile, defining a minimum and maximum of the time range within each participant had to react in order to win money (grey area = time range 0 to 1). Due to the fact that these ranges tended to be low (~5 to 10 milliseconds), we dispersed the time frame, ranging from -2.5 ranges below the original minimum and 1 range above the original maximum. Finally, the pay-out amount was determined by plotting the amount won in each respective trial with this modified dispersion of the original time range. With respect to the pay-out structure this approach allowed the task to possess a “realistic feel” by providing more dispersed outcome amounts. The X-axis represents the corresponding percentage of the maximal possible win during each trial. For example: in order to win 40% of the maximum amount during the CHF 2 condition (40%=CHF 0.8), participants had to react -1 ranges below the minimum. In order to win 80% (CHF 1.6), the response time had to be 1 range above the minimum.

1. Supplementary Results
   1. Table S1. Whole brain analyses of reward coding regions across all subjects

|  | X | Y | Z | Cluster Size | t |
| --- | --- | --- | --- | --- | --- |
| Middle frontal gyrus | -24 | 24 | 52 | 1780 | 6.29 |
|  | -17 | 18 | 61 |  | 5.44 |
|  | -22 | 43 | 25 |  | 5.43 |
| Inferior parietal lobe | -46 | -63 | 40 | 7494 | 6.29 |
|  | 16 | -88 | 25 |  | 6.07 |
|  | 6 | -75 | 24 |  | 5.70 |
| Caudate | 25 | -31 | 28 | 3062 | 6.07 |
|  | 30 | -18 | 28.5 |  | 5.35 |
|  | 16 | -33 | 69 |  | 5.33 |
| Insula/ inferior frontal gyrus | 60 | 3 | 12 | 657 | 5.84 |
|  | 31 | -6 | 3 |  | 5.80 |
|  | 39 | -4 | 10 |  | 4.38 |
| Putamen | -21 | 6 | -10 | 1300 | 5.5 |
|  | -9 | 24 | -16 |  | 5.1 |
|  | -23 | 12 | -2 |  | 5.0 |
| Putamen | 23 | 6 | -10 | 685 | 5.4 |
|  | 8 | 23 | -11 |  | 4.5 |
|  | 14 | 21 | -2 |  | 4.3 |
| mOFC | -8 | 54 | -6 | 267 | 5.0 |
|  | -3 | 62 | 1 |  | 4.2 |
| Postcentral gyrus | -26 | -36 | 73 | 781 | 4.8 |
|  | -24 | -28 | 54 |  | 4.4 |
|  | -22 | -30 | 67 |  | 4.4 |
| Paracentral gyrus | 5 | -31 | 61 | 830 | 4.7 |
|  | -2 | -43 | 60 |  | 4.4 |
|  | -6 | -40 | 51 |  | 4.3 |
| Voxel-wise whole brain analysis of the contrast reward outcome [(pmod low reward) + (pmod high reward)], across all subjects FWE-corrected p<0.05. | | | | | |

- 1. Table S2. Within-group analysis of adaptive coding in healthy controls

|  | X | Y | Z | cluster size | t |
| --- | --- | --- | --- | --- | --- |
| Paracentral Lobule | 5 | -30 | 66 | 253 | 8.4 |
|  | 5 | -32 | 62 |  | 7.1 |
| Precuneus/Cuneus | 18 | -87 | 26 | 633 | 7.6 |
|  | 12 | -90 | 17 |  | 6.0 |
|  | 14 | -93 | 12 |  | 5.7 |
|  | 24 | -93 | 9 |  | 4.9 |
|  | 9 | -84 | 32 |  | 4.3 |
|  | 23 | -80 | 36 |  | 3.7 |
|  | 20 | -78 | 29 |  | 3.5 |
| Caudate | 21 | 3 | 23 | 108 | 5.7 |
|  | 20 | -3 | 24 |  | 4.8 |
|  | 20 | -7 | 24 |  | 4.7 |
| Cingulate Cortex | 24 | -35 | 29 | 121 | 5.7 |
|  | 23 | -42 | 32 |  | 4.4 |
| Superior Frontal Gyrus | -20 | 24 | 54 | 99 | 5.6 |
|  | -24 | 23 | 53 |  | 5.0 |
|  | -17 | 30 | 54 |  | 3.7 |
| Insula | 50 | -3 | 6 | 98 | 5.3 |
|  | 45 | -4 | 9 |  | 4.9 |
|  | 39 | -7 | 12 |  | 4.2 |
|  | 42 | 0 | 12 |  | 3.9 |
| Precentral Gyrus | 63 | 5 | 12 | 87 | 5.2 |
| Precuneus/Cuneus | 3 | -71 | 23 | 130 | 4.9 |
|  | 6 | -68 | 21 |  | 4.2 |
|  | 5 | -75 | 26 |  | 4.1 |
| Middle Frontal Gyrus | -27 | 38 | 38 | 177 | 4.8 |
|  | -21 | 39 | 45 |  | 4.7 |
|  | -21 | 39 | 36 |  | 4.6 |
|  | -24 | 39 | 42 |  | 4.3 |
|  | -20 | 42 | 33 |  | 4.2 |
|  | -18 | 48 | 35 |  | 3.7 |
| Caudate | -26 | -4 | 26 | 116 | 4.6 |
|  | -18 | 3 | 29 |  | 4.3 |
|  | -20 | -4 | 26 |  | 4.1 |
|  | -17 | 3 | 24 |  | 3.8 |
| Postcentral Gyrus | 11 | -36 | 71 | 96 | 4.6 |
|  | 18 | -30 | 63 |  | 4.2 |

Significant activation in the adaptive coding contrast [(pmod low reward)-(pmod high reward)] within the previously identified reward coding brain regions, across healthy controls FWE-corrected p<0.05. In patients with schizophrenia, we did not find any significant activation in the adaptive coding contrast.

1. References

1 Kirkpatrick B, Strauss GP, Nguyen L, Fischer BA, Daniel DG, Cienfuegos A *et al.* The brief negative symptom scale: psychometric properties. *Schizophr Bull* 2011; **37**: 300–5.

2 Addington D, Addington J, Schissel B. A depression rating scale for schizophrenics. *Schizophr Res* 1990; **3**: 247–51.

3 Frances A, Pincus HA, First MB. The Global Assessment of Functioning Scale (GAF). Diagnostic and Statistical Manual of Mental Disorders. 4th ed. Washington, DC: 1994.

4 Juckel G, Schaub D, Fuchs N, Naumann U, Uhl I, Witthaus H *et al.* Validation of the Personal and Social Performance (PSP) Scale in a German sample of acutely ill patients with schizophrenia. *Schizophr Res* 2008; **104**: 287–293.

5 Helmstaedter C, Lendt M, Lux S. VLMT. Verbaler Lern- und Merkfähigkeitstest. 2001.

6 Härting C, Markowitsch HJ, Neufeld H. Wechsler Memory Scale - Revised Edition, German Edition. Manual. Huber: Bern, Switzerland, 2000.

7 Kessels RP, van Zandvoort MJ, Postma A, Kappelle LJ, de Haan EH. The Corsi Block-Tapping Task: standardization and normative data. *Appl Neuropsychol* 2000; **7**: 252–258.

8 Von Aster M, Neubauer A, Horn R. *Wechsler Intelligenztest für Erwachsene WIE. Deutschsprachige Bearbeitung und Adaption des WAIS-III von David Wechsler*. Pearson Assessment; 2006: Frankfurt, Germany, 2006.

9 Shallice T. Specific impairments of planning. *Philos Trans R Soc Lond B Biol Sci* 1982; **298**: 199–209.

10 Delis DC, Kaplan E, Kramer J. *Delis Kaplan Executive Function System*. The Psychological Corporation; 2001: San Antonio, TX, 2001.

11 Lehrl S, Triebig G, Fischer B. Multiple choice vocabulary test MWT as a valid and short test to estimate premorbid intelligence. *Acta Neurol Scand* 1995; **91**: 335–345.

12 Kirschner M, Hager OM, Bischof M, Hartmann MN, Kluge A, Seifritz E *et al.* Ventral striatal hypoactivation is associated with apathy but not diminished expression in patients with schizophrenia. *J Psychiatry Neurosci JPN* 2015; **40**: 140383.
